# Cardiovascular Disease in Chronic Kidney Disease: Implications of Cardiorespiratory Fitness, Race, and Sex

**DOI:** 10.31083/j.rcm2510365

**Published:** 2024-10-11

**Authors:** Jared M. Gollie, Gauranga Mahalwar

**Affiliations:** ^1^Research and Development, Washington DC VA Medical Center, Washington, DC 20422, USA; ^2^Health, Human Function, and Rehabilitation Sciences, The George Washington University, Washington, DC 20052, USA; ^3^Department of Internal Medicine, Cleveland Clinic Foundation, Cleveland, OH 44195, USA

**Keywords:** chronic kidney disease, fitness, exercise, kidney insufficiency, oxygen consumption, aerobic capacity

## Abstract

Cardiovascular disease (CVD) poses a major health burden in adults with chronic kidney disease (CKD). While cardiorespiratory fitness, race, and sex are known to influence the relationship between CVD and mortality in the absence of kidney disease, their roles in patients with CKD remain less clear. Therefore, this narrative review aims to synthesize the existing data on CVD in CKD patients with a specific emphasis on cardiorespiratory fitness, race, and sex. It highlights that both traditional and non-traditional risk factors contribute to CVD development in this population. Additionally, biological, social, and cultural determinants of health contribute to racial disparities and sex differences in CVD outcomes in patients with CKD. Although cardiorespiratory fitness levels also differ by race and sex, their influence on CVD and cardiovascular mortality is consistent across these groups. Furthermore, exercise has been shown to improve cardiorespiratory fitness in CKD patients regardless of race or sex. However, the specific effects of exercise on CVD risk factors in CKD patients, particularly across different races and sexes remains poorly understood and represent a critical area for future research.

## 1. Introduction

Chronic kidney disease (CKD), now the tenth leading cause of death globally, affects over 850 million people [1, 2]. The actual prevalence of CKD may be even higher, as is it estimated that 40% of adults with severely reduced kidney function are unaware of their condition [3]. Cardiovascular disease (CVD) represents the primary cause of mortality among individuals with kidney disease, underscoring the importance managing cardiovascular risks this patient population [4, 5, 6]. Notably, individuals with moderate-to-severe CKD are at greater risk of mortality from CVD than of progressing to kidney failure that would necessitate kidney replacement therapies [4, 5]. In more advanced stages of CKD (i.e., stages 4 and 5), at least half of all patients have CVD with cardiovascular mortality accounting for approximately 50% of all deaths. CKD is also an independent predictor of myocardial infarction, stroke, and death among males under 55 and females under 65 [7]. The risk of death, cardiovascular events, and hospitalizations increase in a graded fashion and is independently associated with declines in kidney function below estimated glomerular filtration rates (GFR) of 60 mL/min per 1.73 m^2^ and higher albuminuria [8, 9, 10].

Cardiorespiratory fitness, race, and sex are known to influence the relationship between CVD and mortality in individuals without kidney disease [11, 12, 13, 14, 15]. However, the impact of these factors on the relationship between CVD and mortality in patients with CKD remains unclear. Thus, this narrative review aims to discuss the most up-to-date evidence regarding CVD in patients with CKD, with a particular focus on cardiorespiratory fitness, race, and sex.

## 2. Literature Search Strategy

PubMed and Google Scholar were the primary databases used for completing the literature search. Key terms used in the search strategy included “kidney diseases”, “renal failure”, “chronic kidney disease”, “kidney insufficiency”, “aerobic capacity”, “oxygen uptake”, “oxygen consumption”, “aerobic exercise”, “endurance exercise”, “exercise”, “gender”, “sex”, “race”, and “ethnicity”. Most recent publications were prioritized with the inclusion of seminal work when appropriate. Literature searches were conducted from September 2023–January 2024.

## 3. Diagnosing CKD Severity

The Kidney Disease Improving Global Outcomes (KDIGO) defines CKD as abnormalities of kidney structure or function present for more than 3 months with health implications [16]. Staging of CKD is based on the cause of kidney function decline, GFR category, and albuminuria category [16]. The stages of CKD based on GFR encompass normal or high kidney function (G1, GFR ≥90 mL/min/1.73 m^2^) to kidney failure (G5, GFR ≤15 mL/min/1.73 m^2^). Persistent albuminuria categories range from normal to mildly increased (A1, <30 mg/g, <3 mg/mmol) to severely increased (A3, >300 mg/g, >30 mg/mmol). The progression of CKD is determined by assessing GFR and albuminuria at a minimum annually in individuals with established CKD, with more frequent assessments in those at higher risk of progression and/or where measurement will impact treatment decisions [16]. The prevalence of CKD is greatest in those 65 years of age and older, with diabetes and hypertension being the two leading causes of CKD, accounting for three out of every four new cases reported [3].

## 4. Pathophysiology of CVD in Patients with CKD

**Fig. 1.**
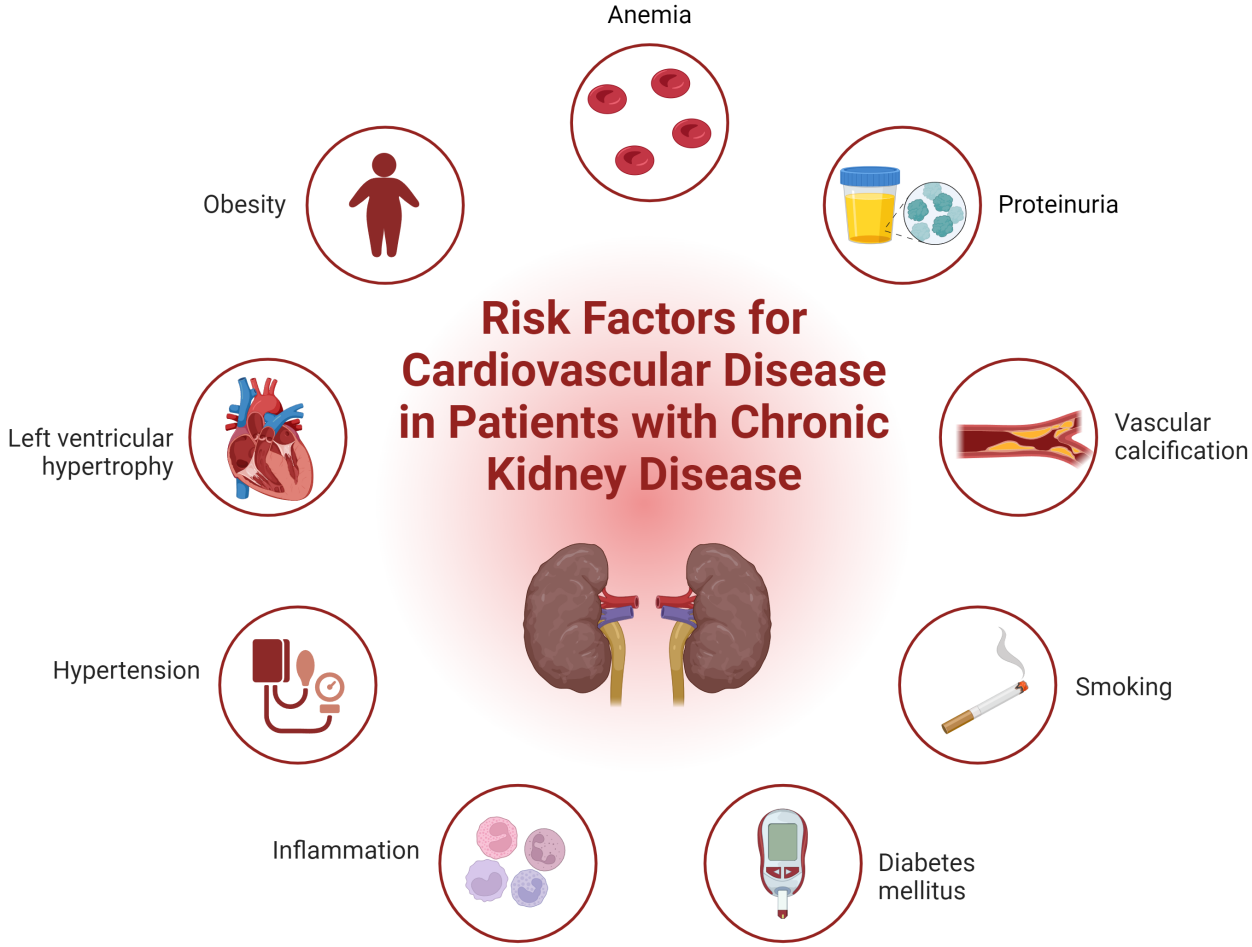
**Integrated overview of cardiovascular risk factors in chronic kidney disease**. Fig. 1 illustrates both traditional and non-traditional risk factors contributing to cardiovascular disease in patients with chronic kidney disease. Traditional risk factors include hypertension, diabetes mellitus, obesity, dyslipidemia, and smoking. Non-traditional risk factors specific to CKD such as albuminuria, altered bone metabolism, systemic inflammation, anemia, hypervolemia, oxidative stress, left ventricular hypertrophy, and the accumulation of toxic metabolites are also depicted. This comprehensive visualization highlights the complex interplay of factors that exacerbate cardiovascular morbidity in CKD patients. CKD, chronic kidney disease. Created with BioRender.

Kidney disease and CVD are intricately connected. Both GFR and albuminuria are independently associated with increased cardiovascular mortality [17]. As CKD progresses, patients commonly develop cardiovascular complications, including atherosclerosis, arterial stiffening, calcification, and cardiomyopathy [18]. Traditional risk factors such as hypertension, diabetes mellitus, obesity, dyslipidemia, and smoking are well-recognized [6, 19]. However, CKD introduces additional risk factors including albuminuria, altered bone metabolism, systemic inflammation, anemia, hypervolemia, increased oxidative stress, left ventricular hypertrophy (LVH), and toxic metabolites, all of which contribute to the pathogenesis (Fig. 1) [6, 19]. These factors promote accelerated senescence of both peripheral blood cells and vascular cells [20, 21]. Furthermore, the atherosclerotic process is exacerbated by increased recruitment of monocytes transform into macrophages and foam cells, the mobilization of vascular smooth muscle cells, and the development of atheroma, ultimately leading to increased cardiovascular morbidity [22]. In later stages of CKD, patients are more likely to present with acute coronary syndrome than with stable ischemic heart disease [23].

Traditional risk factors including hypertension and hyperglycemia, are known to increase the risk of both micro and macrovascular diseases [24]. Dyslipidemia in CKD is characterized by elevated levels of triglycerides and low-density lipoproteins cholesterol, alongside reduced high-density lipoproteins levels [25]. Furthermore, the vaso-protective function of HDL is diminished in patients with CKD in the presence of uremic toxins due to post translational modification [26]. Vascular calcification, both intimal and medial, often results from disrupted bone mineral metabolism in CKD, leading to elevated phosphate levels, increased fibroblast growth factor 23 (FGF 23), and decreased 1,25-dihydroxy vitamin D, all of which can accelerate CVD [22]. This calcification of central arterial vessels contribute to increased pulse wave velocity and cardiac afterload, increasing the risk of heart failure [27, 28, 29]. Concurrently, hyperphosphatemia and elevated FGF 23 levels further contribute to the development of LVH and reduced coronary perfusion, compounding cardiovascular risk [30, 31, 32].

As kidney disease progresses, pro-inflammatory processes such as oxidative stress and reduced clearance of inflammatory cytokines (including C-reactive protein, IL-6 and tumor necrosis factor), intensify, alongside complications from uremia, insulin resistance, and infection [33, 34]. In advanced stages of CKD, patients often exhibit a specific cardiac condition known as uremic cardiomyopathy, characterized by ventricular fibrosis, which contributes to LVH and diastolic dysfunction [35, 36]. Circulatory changes resulting from impaired tubuloglomerular feedback and increased renal sodium resorption can further impact the cardiovascular morphology [35, 36]. Sympathetic overactivity from renin-angiotensin aldosterone system (RAAS) and low bioavailability of nitric oxide can also exacerbate hypertension and kidney dysfunction, increasing the risk for CVD [37, 38, 39]. Anemia due to chronic inflammation and reduced erythropoietin production in patients with CKD further promotes cardiac remodeling [40]. Moreover, the escalation of oxidative stress from increased production and impaired clearance of reactive oxygen species, coupled with enhanced oxidation of lipids, proteins, and DNA combined with the weakening of the antioxidant system contributes to not only development of CVD but overall mortality of patients with CKD [41, 42, 43, 44].

## 5. Role of Race/Ethnicity and Sex in CVD in Patients with CKD

### 5.1 Race

Racial disparities in the prevalence and progression of CKD, as well as the associated mortality from CKD are risk, are well documented [3, 45, 46, 47, 48, 49, 50, 51, 52]. According to the Centers for Disease Control and Prevention (CDC), non-Hispanic black patients exhibit the highest CKD prevalence in the United States at 19.5%, followed by non-Hispanic Asians and Hispanic at 13.7% each, and non-Hispanic whites at 11.7% [3]. Studies also show that black patients are more likely to progress to advanced stages of CKD compared to white patients [45, 46]. Analysis from the Framingham and Framingham Offspring datasets indicates the incidence of cardiac and mortality events per 1000 person-years attributed to kidney disease is significantly higher among African American males (16.1 and 40.5, respectively) compared to white males (4.3 and 13.7, respectively), and similar disparities exist between African American females (13.6 and 14.2, respectively) compared to white females (1.2 and 5.8, respectively) [47]. In contrast to the Framingham dataset findings, black patients have a lower risk of mortality following the initiation of dialysis as well as while on dialysis than their white counterparts [48, 49, 50]. While the mechanisms for these mortality differences are not fully understood and are likely multifactorial, inflammation and elevated levels of interleukin-6 (IL-6) have been identified as contributing factors [51, 52]. Additionally, the relatively younger age of black patients at the initiation of dialysis may partly explain these observed discrepancies [50].

In addition to biological factors, social determinants of health including socioeconomic status, psychosocial factors, healthcare access, neighborhood, and environment significantly contribute to racial disparities in CKD outcomes [53]. This has led to leading organizations re-considering how race is used in the diagnosis of CKD. In a notable 2021 joint statement, the American Society of Nephrology and the National Kidney Foundation advocated for the exclusion of race modifiers from equations previously used to estimate kidney function [54]. Similarly, the American Heart Association’s (AHA) recently published PREVENT (Predicting Risk of cardiovascular disease EVENTs) equation now emphasizes social determinants of health, including the patient’s zip code (to determine patient’s social deprivation index) instead of race, while still considering the patient’s GFR to estimate the 10 to 30-year risk of total CVD [55].

### 5.2 Sex

Genetic, hormonal, behavioral, societal, and cultural factors may contribute to the observed sex differences in CKD progression and cardiovascular outcomes [56, 57]. Females are reported to have a higher prevalence of CKD compared to males (~14% vs ~12%) [3]. Despite this higher prevalence, fewer females undergo dialysis, are less likely to initiate dialysis with arteriovenous fistula, and although more females serve as living kidney donors, they receive fewer kidney transplants compared to their male counterparts [58]. The lower risk for all-cause and cardiovascular mortality, slower CKD progression, and less rapid decline in GFR observed in females versus males may be partially attributed to lower levels of proteinuria [59, 60]. Additionally, older females (at or over 65 years) with CKD at stages 4 or 5 exhibit a lower risk of major adverse cardiovascular events, a relationship that diminishes when adjusting for pre-existing cardiometabolic comorbidities and cardiovascular risk factors [61]. However, non-cardiovascular mortality rates are higher among younger females (under 45 years) and diabetic females with CKD initiating dialysis when compared to males [62].

It is suggested that female sex hormones may confer cardiovascular and renoprotective benefits in females without pre-existing CKD by reducing fibrosis, inflammation, oxidative stress, and modulating RAAS [63, 64, 65]. This protective effect of female sex hormones against CVD is supported by data demonstrating an increased risk for CVD and cardiovascular-related adverse events in females following menopause and those experiencing early menopause [65, 66, 67]. Menopause is generally found to occur earlier in females with end-stage kidney disease (ESKD) primarily due to surgical causes which may place them at an elevated risk for CVD [68]. Interestingly, both higher and lower levels of estradiol are found to be associated with greater risk of cardiovascular mortality in females with ESKD [69, 70].

In male patients with CKD, low endogenous testosterone concentrations are inversely associated with endothelial dysfunction and increased risk of both CVD mortality and all-cause mortality [71, 72]. These findings highlight the complex role of hormones in kidney health and cardiovascular outcomes, underscoring the necessity for further research to elucidate their precise mechanisms of action. Further research into the potential use of hormone replacement therapy in both female and males is required before such therapies can be recommended for addressing CVD in the CKD population. To date, the inclusion of females in clinical trials remains significantly lower than that of males, limiting our understanding of CKD and cardiovascular outcomes in women. Future studies should focus on increasing the recruitment of female participants across various age groups, which is crucial for advancing our knowledge of pathophysiological distinctions between males and females.

## 6. Cardiorespiratory Fitness in Patients with CKD

Cardiorespiratory fitness is a strong predictor of major adverse cardiovascular events, premature mortality, and CKD incidence [73, 74, 75, 76, 77, 78, 79, 80, 81, 82]. Studies demonstrate that higher fitness levels are associated with a reduced relative risk of mortality in both males and females [83, 84]. Specifically, females with high fitness levels experience a 28%, 34%, and 34% lower risk of all-cause, CVD, and cancer mortality, respectively, compared to females with low fitness levels [84]. Similarly, males with high fitness levels have a 54%, 49%, and 46% lower risk of all-cause, CVD, and cancer mortality, respectively, when compared to males with low fitness levels [84]. Cardiorespiratory fitness is shown to be predictive of CKD incidence [73, 74]. Over a median follow-up of 7.9 years, the incidence of CKD is found to be inversely related to exercise capacity [74]. Individuals classified as moderately or highly fit have a 24% and 34% lower risk of developing CKD, respectively, when compared to those classified as low fit [73]. Additionally, every 1-minute reduction in treadmill walking duration is associated with 1.14-fold higher risk of CKD [85]. Despite a higher propensity for developing CKD among black adults, adjusting for fitness level significantly reduces this risk when compared to white adults [86], suggesting that enhanced cardiorespiratory fitness mitigates the excess risk of developing CKD in black adults [85].

### 6.1 Central Limitations in Patients with CKD

Cardiorespiratory fitness is often compromised in patients with CKD and progressively declines as the disease severity increases [87, 88]. In patients with stages 2–5 CKD not on dialysis, aerobic capacity correlates with stroke volume, peak heart rate, and hemoglobin levels [89]. Notably, ESKD patients have lower peak oxygen consumption (VO_2_ peak) compared to those with hypertension [90]. Independent predictors of VO_2_ peak in the ESKD cohort included left ventricular filling pressure and pulse wave velocity, whereas in hypertensive adults, significant predictors also encompass left ventricular mass, left ventricular end-diastolic volume index, peak heart rate, and pulse-wave velocity [90]. These findings suggest that maladaptive left ventricular changes and blunted chronotropic response are important mechanistic factors influencing decreased cardiovascular reserve in ESKD patients [90]. Comparative analysis shows that kidney transplant recipients (KTR) possess higher left ventricular mass and lower left ventricular ejection fraction than hypertensive patients [91]. Of note, VO_2_ peak is shown to improve following kidney transplantation, the results of which seem to occur due to increases in peak heart rate and left ventricular ejection fraction [91, 92]. In a 10-year longitudinal study on ESKD patients highlighted a greater prevalence of myocardial ischemia, left arterial size, thicker anteroseptal walls, and lower VO_2_ peak and heart rate among ESKD patients who died [93]. Moreover, myocardial ischemia and VO_2_ peak were independent predictors of 10-year all-cause mortality [93]. In CKD and ESKD patients with heart disease, VO_2_ peak in those with a GFR below 45 mL/min per 1.73 m^2^ was influenced by left ventricular ejection fraction and hemoglobin levels, while in those with a GFR of 45–59, the end-tidal oxygen partial pressure was the strongest predictor of VO_2_ peak [94]. The initiation of dialysis is also associated with impairments in peak oxygen consumption, with Arroyo *et al*. [95] reporting reduced peak workload, decreased peak heart rate, reduced circulatory power, and increased left ventricular mass index in patients who had been on dialysis vintage for less than 12 months.

### 6.2 Peripheral Limitations in Patients with CKD

Central factors influence oxygen delivery, whereas peripheral factors determine how effectively this oxygen is utilized for energy synthesis during muscle contraction. In a recent systematic review and meta-analysis, Chinnappa *et al*. [96] reported that arterio-venous oxygen (a-vO_2_) difference was a primary contributor to VO_2_ peak in patients with CKD. At peak exercise, hemodialysis patients achieved only 48%, 80%, 73%, and 72% of expected values in VO_2_ peak, cardiac output, heart rate, and a-vO_2_ difference, respectively [97]. Furthermore, during constant load exercise, the relative increases in a-vO_2_ difference and heart rate are lower in ESKD patients compared to age- and sex-matched controls despite exercise being performed at a higher percentage of their maximal minute ventilation and heart rate [98]. Additionally, decreased mechanical efficiency in well-trained KTR, indicated by an increased VO_2_-treadmill speed relationship despite normal VO_2_ peak and heart rate, suggests that aerobic capacity is limited by peripheral factors in this patient population [99]. Taken together, these findings suggest that reduced VO_2_ peak in the CKD population may result from both central and peripheral factors.

## 7. Role of Race, Ethnicity, and Sex in Cardiorespiratory Fitness

### 7.1 Race and Ethnicity

A recent systematic review identified both race/ethnicity and male sex as independent factors influencing cardiorespiratory fitness [100]. Data from the National Health and Nutrition Examination Survey (1999–2004) revealed that cardiorespiratory fitness was significantly higher in Mexican American (40.9 mL/kg/min) and non-Hispanic white adults (40.3 mL/kg/min) compared to non-Hispanic black adults (37.9 mL/kg/min) aged 18–49 years [101]. Race remained a significant independent predictor of cardiorespiratory fitness after adjusting for vigorous intensity physical activity and overall physical activity [101]. Furthermore, using data from the Dallas Heart Study, Pandey *et al*. [102] reported that black adults had the lowest cardiorespiratory fitness (26.3 mL/kg/min) relative to white (29 mL/kg/min) and Hispanic (29.1 mL/kg/min) adults. However, multivariate analysis showed that differences for black adults were attenuated and no longer significantly different from Hispanic adults after adjustments for age, sex, body mass index (BMI), lifestyle factors, socio-economic status, and cardiovascular risk factors [102]. It has been suggested that non-Hispanic blacks adults may be predisposed to reduced aerobic capacity by way of muscle fiber type (i.e., greater percentage of type II muscle fibers) [103]. Despite these differences in fitness among racial and ethnic groups, the importance of cardiorespiratory fitness for cardiovascular health remains consistent across all groups. In a study assessing exercise capacity over approximately seven years, cardiorespiratory fitness was found to be a strong predictor of all-cause mortality among both black and white male veterans with and without CVD [104]. This relationship was inversely graded, showing a similar impact on mortality outcomes between black and white adults [104].

### 7.2 Sex

Typically, the maximal oxygen consumption (VO_2_ max) is about 10% lower in female adults than in their male counterparts [105, 106]. This difference is primarily due to females having a higher percent body fat and lower total hemoglobin mass for a given body weight, which reduces their oxygen-carrying capacity [105, 106, 107]. However, when comparing changes in cardiorespiratory fitness with age, male adults experience a greater decline in VO_2_ max over time compared to females [108]. The relative contributions of maximal cardiac output and maximal a-vO_2_ difference to the decline in VO_2_ max on the other hand appear to be similar between sexes [108]. Importantly, both female and male adults demonstrate similar improvements in lean body mass, VO_2_ max, blood volume, stroke volume, and widening of a-vO_2_ difference following chronic aerobic exercise training [109].

## 8. Physical Activity and Exercise Recommendations for Cardiovascular Health in Patients with CKD

### 8.1 Aerobic Exercise

Engaging in sufficient levels of physical activity is essential for maintaining and improving cardiovascular health and cardiorespiratory fitness. Current guidelines recommend that older adults engage in moderate intensity aerobic exercise at least five days per week, vigorous intensity aerobic exercise at least three days per week, or a combination of moderate and vigorous intensity aerobic activities 3–5 days per week (Table 1) [110]. Moderate-intensity aerobic exercise attenuates age-induced deterioration of the myocardium, improves skeletal muscle blood flow, enhances myocardial perfusion, improves endothelial function, and increases cardiorespiratory fitness, and enhances blood lipids and hemodynamics [111]. For patients with CKD, the recommendations for aerobic exercise are similar to those of older adults with the exception of vigorous intensity activities [110]. Aerobic exercise, whether performed independently or in combination with resistance training, has been shown to be effective for maintaining and improving cardiorespiratory fitness in patients with CKD [112, 113, 114, 115, 116, 117].

**Table 1.** **Aerobic exercise recommendations [110]**.

	Older adults	Chronic kidney disease
Frequency	≥5 d·wk^-1^ for moderate intensity; ≥3 d·wk^-1^ for vigorous intensity; 3–5 d·wk^-1^for a combination of moderate and vigorous intensity	3–5 d·wk^-1^ of moderate intensity
Intensity	Moderate intensity: %$\dot{V}$O_2max_, 40–59, %HR_max_, 64–76, RPE 12–13 on a scale of 6–20; vigorous intensity: %$\dot{V}$O_2max_, 60–89, %HR_max_, 77–95, RPE 14–17 on a scale of 6–20	Moderate intensity (40%–59% $\dot{V}$O_2_R, RPE 12–13 on a scale of 6–20)
Time	30–60 min·d^-1^ of moderate intensity exercise; 20–30 min·d^-1^ of vigorous intensity exercise; or any equivalent combination of moderate and vigorous intensity exercise; may be accumulated over the course of the day	20–60 min of continuous activity; however, if this cannot be tolerated, use 3–5 min bouts of intermittent exercise aiming to accumulate 20–60 min·d^-1^
Type	Any modality that does not impose excessive orthopedic stress, rhythmic activities using large muscle groups (e.g., walking, cycling, swimming)	Prolonged, rhythmic activities using large muscle groups (e.g., walking, cycling, swimming)

Abbreviations: RPE, rating of perceived exertion; *%$\dot{V}$O_2max_*, percent of maximal oxygen consumption; %HR_max_, percent of maximal heart rate; d·wk^-1^, days per week; $\dot{V}$O_2_R, oxygen consumption reserve; min·d^-1^, minutes per day.

### 8.2 Resistance Training

According to an updated scientific statement by the AHA, resistance training effectively counters both traditional and non-traditional CVD risk factors [118]. Specifically, resistance training is reported to improve blood pressure, lipid profile, glycemic control, and body composition [118]. Furthermore, engaging in resistance exercise regularly can enhance vascular function and structure, decrease inflammation, contribute to increases in cardiorespiratory fitness, and improve sleep quality as well as symptoms of anxiety and depression [118]. Resistance training is associated with a 40–70% reduction in the risk of total CVD events, independent of aerobic exercise; specifically when performed 1–3 times weekly, totaling 1–59 minutes [119]. Additionally, just one hour per week of resistance training is also found to be associated with a lower risk of developing metabolic syndrome over a median follow-up of 4 years, independent of aerobic exercise [120].

For patients with CKD, resistance training recommendations align with those proposed for older adults (Table 2, Ref. [110, 121]). However, given the varied severity of CKD and the complexities of this patient population, the evidence base for specific outcomes is less definitive. For example, very limited information is available regarding the safety and efficacy of power-type resistance training in patients with CKD. Despite these gaps, it is evident that resistance exercise offers significant health benefits to patients with CKD including increases in muscle size, strength, and improved physical functioning [122, 123].

**Table 2.** **Resistance training recommendations [110, 121]**.

	Older adults	Chronic kidney disease
Frequency	≥2 d·wk^-1^	≥2 d·wk^-1^
Intensity	Progressive resistance exercise: Light intensity (i.e., 40%–50% 1-RM) for beginners; progress to moderate-to-vigorous intensity (60%–80% 1-RM); alternatively, moderate (5–6) to vigorous (7–8) intensity on a 0–10 RPE scale.	Moderate intensity (60%–70% estimated from 5-RM or 10-RM); alternatively 5–6 on a 0–10 RPE scale.
	Power training: Light-to-moderate loading (30%–60% 1-RM).	
Time	Progressive resistance exercise: 8–10 exercise involving the major muscle groups; ≥1 set of 10–15 repetitions for beginners; progress to 1–3 sets of 8–12 repetitions for each exercise.	8–10 exercise involving the major muscle groups; 1 set to fatigue or 10–15 repetitions; progress to 2–3 sets.
	Power training: 6–10 repetitions with high velocity.	
Type	Progressive resistance exercise or power training programs or weight-bearing calisthenics, stair climbing, and other strengthening activities that use the major muscle groups.	Progressive resistance exercise, Thera-band, ankle/wrist weights, or weight-bearing calisthenics, stair climbing, and other strengthening activities that use the major muscle groups.

Abbreviations: d·wk^-1^, days per week; 1-RM, one repetition maximum; RPE, rating of perceived exertion.

### 8.3 Effects of Exercise on CVD Risk Factors in Patients with CKD 

The effects of exercise on traditional and non-traditional risk factors for CVD in patients with CKD have not been studied as extensively as cardiorespiratory, strength, and functional outcomes. Evidence from systematic reviews and meta-analyses on the effects of exercise on reductions in blood pressure in patients with CKD have yielded inconclusive results [115, 124, 125, 126, 127, 128]. For example, Thompson *et al*. [126] noted a reduction in systolic blood pressure after 24 weeks of exercise, but this effect dissipated after 48–52 weeks. Conversely, evidence from a systematic review and meta-analysis of thirteen studies focused on weight loss in non-dialysis CKD patients, and found that non-surgical interventions—such as exercise, diet modifications, and/or anti-obesity medications—successfully decreased BMI, proteinuria, and systolic blood pressure while preventing further decline in eGFR [128].

The inconsistent findings regarding the effects of exercise on systolic blood pressure in CKD patients may stem from heterogeneity across study populations and potential study bias. However, the combination of weight loss, particularly fat loss, in combination with exercise may be the most effective strategy for managing blood pressure. Independently, exercise has been shown to decrease BMI, waist circumference, and IL-6 in CKD patients not on dialysis [129]. The combination of exercise with daily caloric restriction and aerobic exercise has also been found to result in decreases in body weight, body fat percentage, and markers of oxidative stress and inflammation [130].

## 9. Conclusions

Cardiovascular complications pose major health burdens to patients with CKD. Both traditional and non-traditional risk factors contribute to CVD outcomes within this group. Moreover, biological and social determinants of health contribute to racial disparities in CKD prevalence and outcomes, while sex hormones underlie the differences between males and females. There are also disparities in cardiorespiratory fitness levels by race and sex, with black adults and females generally exhibiting lower fitness levels than white adults and males. Importantly, the influence of cardiorespiratory fitness level on CVD and cardiovascular mortality risk appears consistent across different racial groups. Exercise improves cardiorespiratory fitness levels in patients with CKD irrespective of race and sex. However, the effects of exercise on risk factors for CVD in patients with CKD are less understood and should be the focus of further investigation.
